# Nonlinear Relationship Between Myeloperoxidase Levels and *Helicobacter pylori* Infection Risk in Chinese Adults: A Population-Based Cross-Sectional Study

**DOI:** 10.3390/jcm14176019

**Published:** 2025-08-26

**Authors:** Junteng Zhou, Qihang Kong, Xiaojing Liu, Yan Huang

**Affiliations:** 1 Health Management Center, General Practice Medical Center, West China Hospital, Sichuan University, Chengdu 610041, China; zhoujunteng@scu.edu.cn; 2 Laboratory of Cardiovascular Diseases, Regenerative Medicine Research Center, West China Hospital, Sichuan University, Chengdu 610041, China; 2022324090006@stu.scu.edu.cn; 3 Department of Cardiology, West China Hospital, Sichuan University, Chengdu 610041, China; 4 State Key Laboratory of Respiratory Health and Multimorbidity, Chengdu 610041, China; 5 Research Laboratory for Prediction and Evaluation of Chronic Diseases in the Elderly, National Clinical Research Center for Geriatric Diseases, Chengdu 610041, China; 6 General Practice Research Institute, West China Hospital, Sichuan University, Chengdu 610041, China

**Keywords:** myeloperoxidase, *Helicobacter pylori*, nonlinear association, inflammation, 14C-urea breath test, cross-sectional study

## Abstract

**Objectives:** This study investigates the nonlinear association between myeloperoxidase (MPO) levels and *Helicobacter pylori* (*H. pylori*) infection risk in Chinese adults, evaluating potential modifiers and clinical implications for infection prevention. **Methods:** An analysis was conducted on cross-sectional data from 15,180 adults who underwent routine health examinations between January and December 2021. *H. pylori* infection was diagnosed using the 14C-urea breath test with a threshold of disintegrations per minute (DPM) ≥ 100. ELISA was used to measure plasma MPO levels. Nonlinear associations were assessed through logistic regression, restricted cubic splines, threshold effect analysis, and subgroup interactions. **Results:** The study identified a U-shaped correlation between MPO levels and the risk of *H. pylori* infection. Compared to the middle tertile (T2: 20.6–31 ng/mL), participants in the lowest (T1: ≤20.6 ng/mL; OR = 1.36, 95% CI: 1.24–1.49) and highest tertiles (T3: ≥31 ng/mL; OR = 1.12, 1.02–1.22) exhibited elevated infection risk after full adjustment (*p* < 0.001). DPM levels were notably elevated in T1 (β = 37.1, 26.66–47.57) and T3 (β = 19.27, 8.81–29.72) relative to T2 (*p* < 0.0001). RCS-based threshold analysis identified a nonlinear inflection at 24.0 ng/mL of MPO, where each additional 1 ng/mL of MPO below this threshold was associated with a reduced infection risk (OR = 0.959, 95% CI: 0.947–0.971), whereas levels above increased the risk (OR = 1.004, 95% CI: 1.002–1.007). This pattern aligned with *H. pylori* breath test values, which mirrored the U-shaped trend across MPO tertiles. Subgroup analyses revealed uniform associations between MPO and *H. pylori* infection risk/DPM across various factors such as age, sex, BMI, and metabolic comorbidities, with all interaction *p*-values exceeding 0.05. **Conclusions:** MPO levels exhibit a robust U-shaped association with *H. pylori* infection risk, independent of anthropometric and metabolic confounders. Monitoring MPO may aid in identifying individuals at bidirectional infection risk, suggesting novel insights into the inflammation–infection interplay. The study’s cross-sectional design limits the ability to establish causal relationships, necessitating further longitudinal research to validate these findings and elucidate their clinical implications.

## 1. Introduction

*Helicobacter pylori* (*H. pylori*) is a Gram-negative bacterium that colonizes the gastric mucosa and is one of the most common bacterial infections worldwide, affecting more than half of the global population [1]. The incidence and prevalence of *H. pylori* infection are particularly high in developing countries [2]. For instance, in China, the infection rate remains elevated among individuals from the general population [3]. Persistent *H. pylori* infection is closely associated with chronic gastritis, peptic ulcers, gastric malignancies, and colorectal cancer [4,5], posing a significant public health burden.

Eradication of *H. pylori* infection can provide long-term protection against gastric cancer in high-risk individuals, particularly those without precancerous gastric lesions at baseline [6]. However, current clinical practices still face challenges in accurately identifying individuals at the highest risk for *H. pylori* infection and its related complications [7]. Therefore, the discovery of novel biomarkers and a deeper understanding of susceptibility mechanisms are crucial for early identification of high-risk individuals and the development of effective intervention strategies. In recent years, increasing evidence has highlighted the critical role of host inflammatory responses in modulating susceptibility to *H. pylori* infection and influencing disease progression risk [8,9]. Consequently, inflammation-related biomarkers may represent a promising direction for the prediction and risk stratification of *H. pylori* infection.

Myeloperoxidase (MPO) is a heme-containing enzyme predominantly produced by myeloid lineage cells which functions as a key modulator of inflammatory processes by generating reactive oxidant species that influence cellular signaling and immune responses [10] and serves as a biomarker of neutrophil-mediated inflammation in various diseases [11]. Notably, *H. pylori* neutrophil-activating protein (HP-NAP) has been shown to promote MPO release from human neutrophils [12], and eradication therapy in *H. pylori*-infected individuals has been reported to affect MPO activity [13]. These findings suggest that *H. pylori* infection may modulate MPO levels. However, the relationship between MPO and *H. pylori* infection risk remains unclear, particularly due to a lack of population-based studies that systematically assess this association. Exploring the potential link between circulating MPO levels and *H. pylori* infection could therefore provide novel insights into individualized risk assessment and preventive strategies.

In this investigation, we performed an extensive cross-sectional study utilizing data from healthy Chinese adults attending regular health check-ups, aiming to systematically explore the relationship between plasma MPO levels and the risk of *H. pylori* infection. Specifically, we examined whether the association between MPO levels and *H. pylori* susceptibility follows a linear or nonlinear trend, aiming to clarify its potential biological implications and clinical significance. Our findings may offer epidemiological evidence to support the inflammation–infection interaction hypothesis and lay the groundwork for developing MPO-based predictive tools and individualized prevention strategies for *H. pylori* infection.

## 2. Methods

### 2.1. Study Population

This cross-sectional analysis was based on adult participants who underwent routine health check-ups at West China Hospital, Sichuan University (January–December 2021), as previously reported [14]. Participants met prespecified eligibility criteria: (1) age ≥ 18 years; (2) completion of *H. pylori* infection test; (3) valid MPO measurement; and (4) full anthropometric/laboratory profiling. After sequential exclusions for missing *H. pylori* infection test (*n* = 2590), absent MPO data (*n* = 10), age < 18 years (*n* = 30), incomplete anthropometric (*n* = 94) or laboratory indices (*n* = 1808), prior gastrectomy (*n* = 1), cancer (*n* = 144), and chronic heart disease (*n* = 63), the final cohort comprised 15,180 participants. The study was approved by the Ethics Committee of West China Hospital (Approval No. 2018-303), and written informed consent was obtained from all participants prior to enrollment.

### 2.2. Demographic and Anthropometric Measurements

Demographic (age, sex), lifestyle (smoking, alcohol use), and clinical covariates were collected through standardized questionnaires and physical examinations. Smoking status was grouped into three categories: never, former, or current. (≥1 cigarette/day for ≥6 months). Alcohol consumption was categorized into three groups: never, former, and current drinkers (defined as consuming more than one drink per week). Body mass index (BMI) was computed as weight in kilograms divided by height in meters squared, while waist-to-hip ratio (WHR) was determined using standardized measurement instruments. Hypertension was defined as systolic/diastolic blood pressure ≥ 140/90 mmHg or antihypertensive medication use; diabetes as fasting glucose ≥ 7.0 mmol/L or hypoglycemic therapy; and hyperlipidemia as triglycerides ≥ 1.7 mmol/L, LDL-C ≥ 3.4 mmol/L, or lipid-lowering drug use.

### 2.3. MPO and Other Laboratory Measurement

After an 8-h overnight fast, venous blood was drawn from the cubital vein into EDTA tubes. Plasma was separated by centrifugation (3000 rpm, 15 min) and stored at −80 °C. All analyses were performed in the accredited clinical laboratory of West China Hospital. Liver function markers (ALT, AST, GGT), lipid profiles (HDL-C, LDL-C) were measured using automated biochemical analyzers (Hitachi 7600, Hitachi High-Technologies Corporation, Tokyo, Japan). Pepsinogen I and II were quantified using ELISA kits (Biohit, Helsinki, Finland; PGI: No. 601010.01CH, PGII: No. 601020.02CN). The pepsinogen ratio (PGR) was calculated by dividing the concentration of pepsinogen I (PGI) by that of pepsinogen II (PGII), serving as a biomarker for gastric mucosal function. Plasma MPO levels were measured using a commercially available enzyme-linked immunosorbent assay (ELISA) kit (EACHY Biotechnology, Suzhou, China; Catalog No. MP180303). All procedures were performed in accordance with the manufacturer’s instructions, which have been validated in our previous studies to ensure reliability and reproducibility of the results [14].

### 2.4. H. pylori Infection Detection

To determine *H. pylori* infection status, all participants underwent the carbon-14 urea breath test (^14C-UBT; Zhonghe Headway Bio-Sci & Tech Co., Shenzhen, China) on the same morning that blood samples were collected. This non-invasive diagnostic method detects the presence of urease activity specific to *H. pylori* and was performed according to standardized protocols to ensure consistency and accuracy across all subjects. Participants ingested a 14C-labeled urea capsule within 2 h post-phlebotomy and provided breath samples 15 min post-administration. Radioactivity was measured using a liquid scintillation counter (LB 2045, Berthold Technologies, Bad Wildbad, Germany). Disintegrations per minute (DPM) values ≥ 100 were classified as *H. pylori*-positive, consistent with standardized diagnostic criteria [15]. This synchronized approach prevented temporal discordance in infection biomarkers.

### 2.5. Statistical Analysis

The presentation of continuous variables is in the form of mean ± SD or median (interquartile range), and categorical variables are given as frequencies. Baseline differences across MPO tertiles were assessed using ANOVA (for normally distributed variables), Kruskal–Wallis tests (non-normal), and χ^2^ tests (categorical variables). Logistic regression models evaluated associations between MPO tertiles (T1, T2, T3) and *H. pylori* infection risk, with T2 as the reference. Three adjustment models were constructed: Model 1 (age, sex); Model 2 (+BMI, WHR, smoking, alcohol); Model 3 (+diabetes, hyperlipidemia, pepsinogen I/II ratio, HDL-C, LDL-C). Linear regression analyzed DPM values with similar adjustments. Restricted cubic splines (RCS) with three knots tested nonlinear relationships, and two-piecewise regression identified inflection points. Subgroup analyses assessed interactions between MPO tertiles and prespecified variables (age, sex, BMI, smoking, alcohol, comorbidities), with multiplicative interaction terms evaluated. All analyses were performed using Free Statistics software (version 2.0; Beijing FreeClinical Medical Technology Co., Ltd., Beijing, China). A two-tailed *p* < 0.05 defined statistical significance.

## 3. Results

### 3.1. Baseline Characteristics of Participants

**Figure 1:**
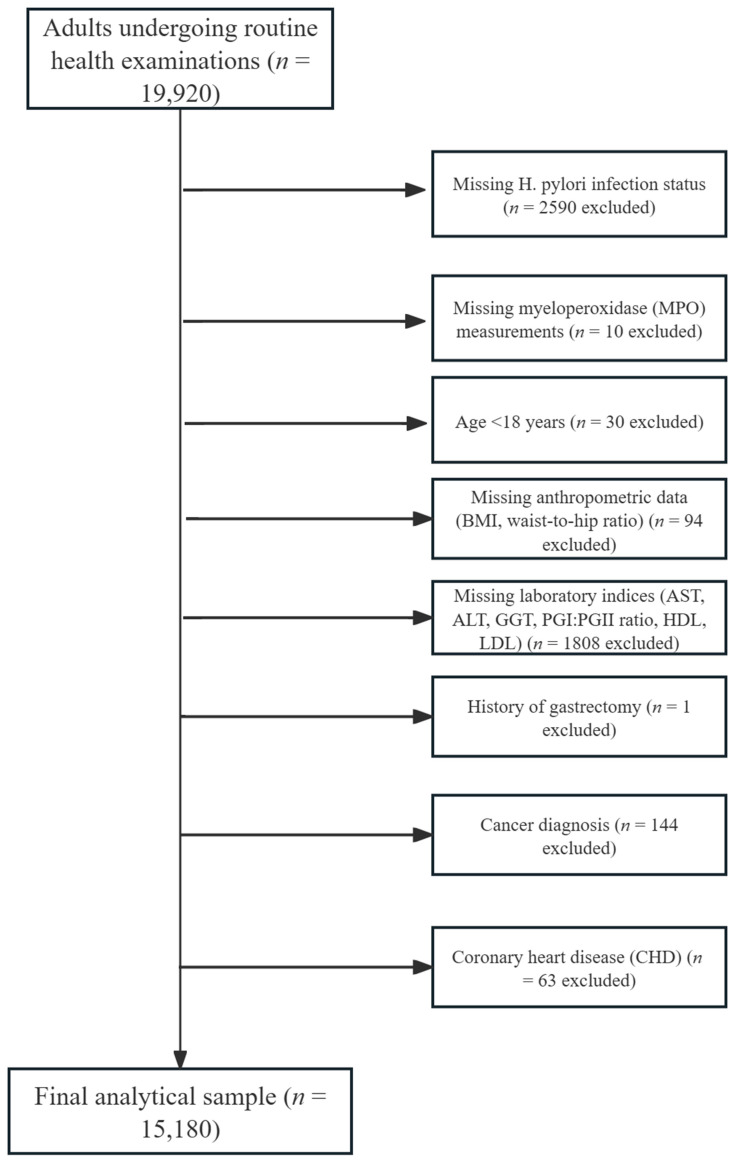
Flowchart of Study Population Inclusion and Exclusion Criteria.

Figure 1 details the cohort selection process. From 19,920 initial examinees meeting inclusion criteria, 15,180 were retained after protocol-driven exclusions: missing *H. pylori* test (*n* = 2590), missing MPO data (*n* = 10), incomplete laboratory data (*n* = 1808), age < 18 years (*n* = 30), anthropometric deficiencies (*n* = 94), and predefined comorbidity exclusions (cancer/chronic heart disease/gastrectomy: *n* = 144 + 63 + 1). The final analytic cohort comprised 15,180 Chinese adults, among whom 4699 (30.96%) tested positive for *H. pylori* infection. Participants were stratified into MPO tertiles: T1 (MPO ≤ 20.6 ng/mL), T2 (20.6–31 ng/mL), and T3 (≥31 ng/mL). Baseline characteristics revealed significant differences across tertiles (Table 1, Figure S1). Participants in the highest tertile (T3) were younger (mean age: 44.3 vs. 46.7 years in T1, *p* < 0.0001), with higher proportions of females (49.4% vs. 45.4% in T1, *p* < 0.001) but lower proportions of current alcohol drinkers (42.5% vs. 47.2% in T1, *p* < 0.0001). Significant group disparities were observed for cardiometabolic traits: waist-to-hip ratio declined steadily from T1 to T3 (0.85 ± 0.08 to 0.84 ± 0.08, *p* < 0.0001), hyperlipidemia prevalence was highest in T1 (1.83% vs. 1.21% in T3, *p* = 0.04), and diabetes rates showed an inverse gradient (T1: 2.93% vs. T3: 1.74%, *p* < 0.001). Hematologic parameters exhibited substantial variations, with T3 showing elevated white blood cell counts (WBC: 6.23 vs. 5.50 × 10^9^/L, *p* < 0.0001), platelet counts (212.13 vs. 198.72 × 10^9^/L, *p* < 0.0001), lymphocyte counts (1.70 vs. 1.53 × 10^9^/L, *p* < 0.0001), neutrophil counts (3.82 vs. 3.22 × 10^9^/L, *p* < 0.0001), and monocyte counts (0.38 vs. 0.32 × 10^9^/L, *p* < 0.0001) compared to T1. Notably, *H. pylori* infection prevalence displayed a U-shaped distribution across MPO tertiles, with the lowest rate in T2 (28.8%) compared to T1 (31.8%) and T3 (32.3%, *p* < 0.001). This pattern mirrored DPM values, which were elevated at both MPO extremes (median [IQR]: T1 = 40.0 [0–160], T3 = 35.0 [0–170] vs. T2 = 25.0 [0–130], *p* < 0.0001). In contrast, liver enzymes (AST, ALT, GGT) showed no statistically significant inter-tertile differences (*p* > 0.05), while LDL-C, HDL-C and PGI/PGII exhibited significant differences (all *p* < 0.05). Supplementary Table S1 compares participants included in the final analysis (*n* = 15,180) with those excluded due to missing data (*n* = 4740). The cohorts demonstrated comparable distributions for age, sex, cardiometabolic indices (BMI, waist–hip ratio), inflammatory biomarkers (MPO, WBC subtypes), and *H. pylori* infection status. Excluded participants had higher burdens of hypertension (12.83% vs. 6.43%) and diabetes (7.07% vs. 2.29%), consistent with our systematic exclusion for chronic heart disease patients and cancer cases.

### 3.2. Association Between MPO Levels and H. pylori Infection/DPM Values

Initial crude logistic regression models revealed a consistent U-shaped pattern linking MPO levels to *H. pylori* infection risk. Compared to the reference tertile (T2), participants in both the lowest (T1) and highest (T3) MPO tertiles exhibited elevated infection odds (T1: OR = 1.15, 95% CI: 1.05–1.25, *p* = 0.002; T3: OR = 1.18, 1.08–1.28, *p* < 0.001; Table 2). This bidirectional trend persisted after sequential adjustment for covariates: in Model 1 (minimally adjusted for age, sex), T1 and T3 retained significantly higher odds (OR = 1.14, *p* = 0.002 and OR = 1.19, *p* < 0.0001, respectively); after adjustment for lifestyle factors in Model 2 (BMI, waist-hip ratio, smoking, alcohol), the associations remained essentially unchanged; and crucially, additional adjustment for metabolic comorbidities and hematologic inflammatory markers in Model 3 amplified the risks for T1 (OR = 1.36, 1.24–1.49, *p* < 0.0001) while maintaining significance for T3 (OR = 1.12, 95% CI: 1.02–1.22, *p* = 0.02). A parallel nonlinear relationship was observed for DPM values: unadjusted analyses showed elevated levels in both T1 (β = 22.45, 95% CI: 11.63–33.27, *p* < 0.0001) and T3 (β = 24.75, 95% CI: 13.91–35.59, *p* < 0.0001), with Model 3 adjustment intensifying this association at low MPO levels (β = 37.1, 95% CI: 26.66–47.53, *p* < 0.0001) while slightly attenuating but retaining significance at high levels (β = 19.27, 95% CI: 8.81–29.72, *p* < 0.001).

To address potential confounding by active inflammation, we performed a targeted sensitivity analysis by excluding all participants with abnormal hematologic inflammation markers (WBC > 9.5 × 10^9^/L, lymphocytes > 3.2 × 10^9^/L, neutrophils > 6.3 × 10^9^/L, or monocytes > 0.6 × 10^9^/L), which removed 945 individuals. Results also demonstrated a U-shaped pattern in this subgroup with normal inflammatory status: for *H. pylori* infection risk in Model 3, T1 showed higher risk (OR = 1.40, 1.27–1.54, *p* < 0.0001) and T3 remained significant (OR = 1.12, 1.02–1.23, *p* = 0.02), while for DPM values, T1 showed markedly higher levels (β = 39.32, 28.53–50.12, *p* < 0.0001) with sustained significance for T3 (β = 18.98, 8.22–29.75, *p* < 0.001; Supplementary Table S2). Notably, despite effect magnitude variations across adjustment stages, the fundamental U-shaped relationship persisted in both primary and sensitivity analyses.

### 3.3. Nonlinear Threshold Effects

**Figure 2:**
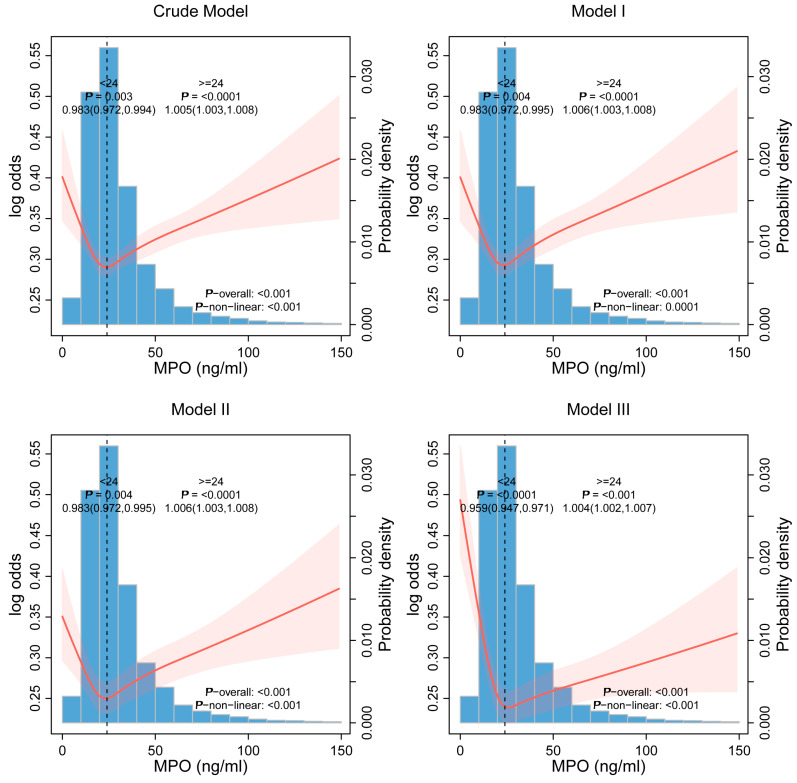
Nonlinear Association Between MPO Levels and *H. pylori* Infection Risk. Model 1: adjust for Age, Sex; model 2: adjust for Age, Sex, BMI, Waist-to-hip ratio, Smoke and Drink; model 3: adjust for Age, Sex, BMI, Waist-to-hip ratio, Smoke, Drink, Diabetes, Hyperlipidemia, PGI/PGII, HDL, LDL, WBC, Platelet, Lymphocyte, Neutrophil and Monocyte.

**Figure 3:**
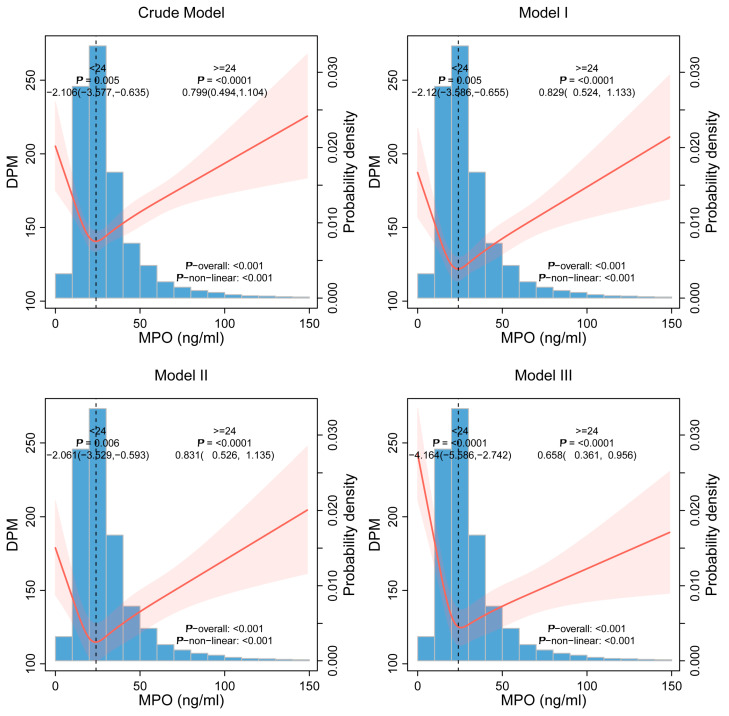
Nonlinear Association Between MPO Levels and DPM Values.

Restricted cubic spline (RCS) analysis revealed a statistically significant nonlinear association between continuous plasma MPO levels and the risk of *H. pylori* infection, displaying a distinct U-shaped pattern. This relationship remained robust, with a *p*-value for nonlinearity of less than 0.001, indicating a departure from linearity. The detailed curve illustrating this association is presented in Figure 2. Using two-piecewise linear regression analysis, we identified a critical inflection point at a plasma MPO concentration of 24.0 ng/mL (Table 3). In the range below this threshold, each 1 ng/mL increment in plasma MPO concentration was associated with an approximate 4.1% (OR = 0.959, 95% CI: 0.947–0.971) reduction in the odds of *H. pylori* infection, whereas levels above 24.0 ng/mL increased risk (OR = 1.004, 95% CI:1.002–1.007, *p* < 0.0001). Similarly, DPM values demonstrated a mirrored pattern (Figure 3): MPO levels < 24.0 ng/mL inversely correlated with DPM (β = −4.164, 95% CI: −5.586 to −2.742, *p* < 0.0001), while higher levels exhibited positive associations (β = 0.658, 95% CI: 0.361–0.956, *p* < 0.0001) (Table 4). The two piecewise models significantly outperformed linear assumptions across all adjustments (log-likelihood ratio *p* < 0.0001).

### 3.4. Subgroup and Interaction Analyses

Stratified analyses revealed no significant interactions between MPO tertiles and *H. pylori* infection risk across Age, Sex, BMI categories, smoking status, alcohol consumption, or metabolic comorbidities (all *p* for interaction >0.05, Table 5). Similarly, DPM values showed consistent associations with MPO tertiles regardless of these subgroups (all *p* for interaction >0.05, Table 6), suggesting the U-shaped relationship remained robust across clinically relevant populations.

## 4. Discussion

To our knowledge, this study is the first large-scale, population-based cross-sectional investigation that systematically explores the association between circulating MPO levels and the presence of *H. pylori* infection in a Chinese adult population after controlling for major confounding factors. The results revealed no linear relationship between MPO levels and the risk of *H. pylori* infection; instead, a significant U-shaped association was observed, indicating that both low and high MPO levels were linked to an increased risk of infection. Moreover, this nonlinear trend was also reflected in the distribution of the delta over baseline (DOB) values from the urea breath test, further supporting the bidirectional risk characteristics between MPO levels and the severity of *H. pylori* infection.

The observed U-shaped association between MPO levels and *H. pylori* infection may be attributable to the biological functions of MPO. On the one hand, MPO possesses potent antimicrobial properties and plays a critical role in innate immunity [16,17]. Previous studies have demonstrated that MPO-deficient neutrophils exhibit impaired bactericidal activity [18]. In addition, significant inhibition of MPO has been shown to upregulate CD11b expression on the surface of neutrophils [19], and CD11b-positive neutrophils are involved in the regulation of *H. pylori*-associated gastric mucosal inflammation [20]. Therefore, low MPO levels may reflect weakened local anti-inflammatory and antimicrobial defenses, impairing the ability to clear *H. pylori* effectively and thereby increasing the risk of persistent infection. Notably, *H. pylori* exhibits resistance to millimolar concentrations of hypochlorous acid, a powerful oxidant generated by MPO [21], which may underlie its ability to colonize the gastric mucosa under specific MPO concentration conditions. However, no study has yet systematically confirmed a direct antimicrobial effect of MPO on *H. pylori*. Further investigation into the concentration-dependent activity of MPO against *H. pylori* is essential to elucidate the biological mechanisms by which the gastric mucosa resists colonization. On the other hand, increased concentrations of MPO have been associated with the development and progression of multiple chronic inflammatory disorders [16]. For example, a prior study showed that iodoacetamide-induced gastritis was associated with increased gastric mucosal injury and elevated local MPO activity [22]. MPO has also been shown to promote the formation of neutrophil extracellular traps (NETs) [23], which have recently been recognized as important mediators of gastric mucosal injury [24]. Moreover, excessive MPO activity may also lead to excessive production of reactive oxygen species [25], which in turn contribute to gastric mucosal damage and disruption of the epithelial barrier [26], potentially facilitating *H. pylori* colonization and infection. Taken together, these findings suggest that both impaired immune defense at low MPO levels and inflammation-induced mucosal injury at high MPO levels may together account for the observed U-shaped risk pattern observed in our study.

Although previous studies have suggested a potential role for MPO in infectious diseases, systematic investigations into its association with *H. pylori* infection risk remain limited. Most existing research has primarily focused on changes in MPO levels in response to established infection. For instance, it has been reported that *H. pylori* neutrophil-activating protein may increase MPO release from neutrophils, contributing to the pathogenesis of *H. pylori*-associated gastritis [12]. In addition, several studies have shown that eradication therapy for *H. pylori* significantly reduces MPO activity [13,27], indicating that MPO may act as a mediator involved in *H. pylori*-related pathologies. However, these studies mainly reflect the inflammatory response after infection and do not address the prospective relationship between baseline MPO levels and the risk of *H. pylori* acquisition. A previous report noted that a functional polymorphism in the MPO gene (G-463A), which leads to reduced MPO expression and activity, was associated with clinical outcomes of *H. pylori* infection [28]. This finding highlights the relevance of inter-individual variability in MPO levels in modulating host response and disease progression following infection. Nonetheless, whether MPO plays a role in susceptibility to *H. pylori* infection itself remains unclear.

Currently, potential biomarkers for *H. pylori* infection and its related diseases are mainly derived from *H. pylori*-associated products and host response-related molecules following stress, including certain non-coding RNAs, DNA damage markers, and H. pylori-specific substances [29]. In contrast, immune-inflammatory molecules have been relatively underexplored. In this study, we assessed the potential of MPO as an independent diagnostic biomarker and found its effectiveness to be limited. The ROC curve analysis revealed an AUC of 0.504, with the optimal threshold identified at 35.728 ng/mL. At this cutoff, the sensitivity was 26.4% while specificity reached 76.4%. The modest discriminative performance may be partially attributed to the U-shaped relationship observed between MPO concentrations and *H. pylori* infection risk, though further studies are needed to confirm this pattern. Nevertheless, MPO could still provide meaningful utility for stratifying risk within the general population. Our analysis demonstrated that individuals falling within both the lowest and highest tertiles of MPO levels showed a roughly 15–18% elevated risk of *H. pylori* infection compared to those in the middle tertile. This pattern suggests that plasma MPO could act as an early warning marker. While it lacks sufficient accuracy for diagnostic purposes, MPO is readily measurable through standard blood tests and may aid in identifying at-risk subpopulations. Therefore, MPO shows promise for use in extensive screening initiatives or as part of a combined biomarker predictive framework.

Even though this extensive population-based study systematically explores the intricate, nonlinear correlation between plasma MPO levels and *H. pylori* infection and offers several notable strengths, certain limitations must be considered. First, the cross-sectional nature of the study precludes any conclusions regarding causality. Future prospective cohort studies are warranted to further assess whether changes in MPO levels can predict the incidence of *H. pylori* infection. Second, MPO levels may be influenced by external factors such as acute inflammatory status, smoking, and dietary habits. Although we adjusted for major confounding variables, residual confounding cannot be entirely ruled out. Third, we only measured plasma MPO levels and did not evaluate local MPO expression in the gastric mucosa. Future studies incorporating tissue-level assessments are necessary to better understand the role of MPO in local immune responses. Furthermore, the generalizability of our results is primarily constrained to urban Chinese populations, as participants were recruited from a tertiary hospital-based health screening program serving predominantly city residents. This reflects intrinsic selection bias toward populations with greater healthcare access-precisely the demographic where *H. pylori* screening interventions are most feasible and clinically prioritized in China’s current healthcare landscape. Future multi-center studies encompassing broader geographic and socioeconomic diversity are warranted to confirm generalizability. While our findings elucidate the nonlinear MPO-*H. pylori* association in urban Southwest Chinese adults—representing populations where guideline-based *H. pylori* screening is clinically prioritized—three considerations merit emphasis. First, the observed metabolic comorbidity imbalance (higher hypertension/diabetes in excluded participants) derives principally from our protocol-specified exclusion of advanced chronic conditions (heart disease, cancer), which inherently concentrate metabolic disorders. Second, this exclusion strategy intentionally enhanced internal validity for primary prevention contexts by removing terminal comorbidities that fundamentally alter inflammatory trajectories. Crucially, the core MPO–infection relationship remained robust across analyses adjusting metabolic diseases, demonstrating stability despite cohort selection. Collectively, while generalizability to rural populations or those with severe multimorbidity requires validation, the results provide clinically actionable insights for *H. pylori* risk stratification in urban preventive healthcare settings where such screening occurs. Future research should integrate longitudinal follow-up, gastric mucosal biopsies, cellular and animal models, and genetic epidemiology approaches to elucidate the functional role and molecular mechanisms of MPO in the onset and progression of *H. pylori* infection. These efforts may also help evaluate the feasibility of MPO-targeted interventions as novel preventive or therapeutic strategies.

In summary, this is the first large-scale cohort study to reveal a U-shaped association between MPO levels and the risk of *H. pylori* infection in a large-scale general population cohort. These findings suggest a dual role of MPO in infection susceptibility and offer new insights into the involvement of inflammatory mediators in *H. pylori* pathogenesis. Our results provide a theoretical basis for developing inflammation-based risk assessment models and personalized prevention strategies. Future multidimensional studies are needed to validate and expand upon these findings and explore the translational potential of MPO in the clinical management of *H. pylori* infection.

## Figures and Tables

**Table 1:** Baseline Characteristics of Participants by MPO Tertiles.

	Total (*n* = 15,180)	T1 <= 20.6 (*n* = 5061)	T2, (20.6, 31] (*n* = 5059)	T3, >=31 (*n* = 5060)	*p* Value
Age	45.66 ± 11.51	46.65 ± 10.98	46.01 ± 11.50	44.31 ± 11.92	<0.0001
Sex					<0.001
Female	7223 (47.58)	2309 (45.42)	2417 (47.92)	2497 (49.43)	
Male	7957 (52.42)	2775 (54.58)	2627 (52.08)	2555 (50.57)	
BMI (kg/m^2^)					0.01
<24	5077 (33.45)	1705 (33.54)	1761(34.91)	1611 (31.89)	
[24, 28)	8709 (57.37)	2936 (57.75)	2821(55.93)	2952 (58.43)	
>=28	1394 (9.18)	443 (8.71)	462(9.16)	489 (9.68)	
Waist-to-hip ratio	0.85 ± 0.08	0.85 ± 0.08	0.85 ± 0.08	0.84 ± 0.08	<0.0001
Smoke					0.07
Current	3546 (23.36)	1219 (23.98)	1157 (22.94)	1170 (23.16)	
Former	667 (4.39)	249 (4.90)	222 (4.40)	196 (3.88)	
Never	10,967 (72.25)	3616 (71.13)	3665 (72.66)	3686 (72.96)	
Drink					<0.0001
Current	6713 (44.22)	2398 (47.17)	2170 (43.02)	2145 (42.46)	
Former	125 (0.82)	36 (0.71)	46 (0.91)	43 (0.85)	
Never	8342 (54.95)	2650 (52.12)	2828 (56.07)	2864 (56.69)	
Hypertension					0.21
No	14,204 (93.57)	4766 (93.75)	4695 (93.08)	4743 (93.88)	
Yes	976 (6.43)	318 (6.25)	349 (6.92)	309 (6.12)	
Diabetes					<0.001
No	14,833 (97.71)	4935 (97.07)	4934 (97.82)	4964 (98.26)	
Yes	347 (2.29)	149 (2.93)	110 (2.18)	88 (1.74)	
Hyperlipidemia					0.04
No	14,944 (98.45)	4991 (98.17)	4962 (98.37)	4991 (98.79)	
Yes	236 (1.55)	93 (1.83)	82 (1.63)	61 (1.21)	
AST(U/L)	21.00 (17.00, 26.00)	21.00 (18.00, 25.00)	21.00 (17.00, 26.00)	21.00 (17.00, 25.00)	0.64
ALT (U/L)	20.00 (14.00, 30.00)	20.00 (14.00, 30.00)	20.00 (14.00, 30.00)	19.00 (14.00, 30.00)	0.28
GGT (U/L)	22.00 (14.00, 38.00)	22.00 (14.00, 38.00)	22.00 (14.00, 37.00)	22.00 (14.00, 37.00)	0.10
PGI/PGII	8.04 ± 2.90	8.49 ± 2.91	7.85 ± 2.78	7.77 ± 2.97	<0.0001
HDL-C (mmol/L)	1.51 ± 0.42	1.50 ± 0.42	1.51 ± 0.41	1.53 ± 0.42	<0.01
LDL-C (mmol/L)	2.96 ± 0.80	3.02 ± 0.81	2.97 ± 0.80	2.90 ± 0.79	<0.0001
WBC (×10^9^/L)	5.82 ± 1.56	5.50 ± 1.36	5.73 ± 1.43	6.23 ± 1.78	<0.0001
Platelet (×10^9^/L)	205.09 ± 60.06	198.72 ± 58.75	204.46 ± 59.06	212.13 ± 61.60	<0.0001
Lymphocyte (×10^9^/L)	1.63 ± 0.59	1.53 ± 0.57	1.65 ± 0.58	1.70 ± 0.60	<0.0001
Neutrophil (×10^9^/L)	3.48 ± 1.23	3.22 ± 1.04	3.41 ± 1.10	3.82 ± 1.44	<0.0001
Monocyte (×10^9^/L)	0.35 ± 0.12	0.32 ± 0.11	0.35 ± 0.12	0.38 ± 0.13	<0.0001
MPO (ng/mL)	31.31 ± 26.98	15.19 ± 3.59	25.32 ± 2.90	53.51 ± 37.10	<0.0001
DPM	35.00 (0.00, 150.00)	40.00 (0.00, 160.00)	25.00 (0.00, 130.00)	35.00 (0.00, 170.00)	<0.0001
*H. pylori* infection					<0.001
No	10,481 (69.04)	3470 (68.25)	3590 (71.17)	3421 (67.72)	
Yes	4699 (30.96)	1614 (31.75)	1454 (28.83)	1631 (32.28)	

BMI, body mass index; AST, aspartate aminotransferase; ALT, alanine aminotransferase; GGT, g-glutamyl transpeptidase; HDL-C, high-density lipoprotein cholesterol; LDL-C, low-density lipoprotein cholesterol; WBC, white blood cell; FPG, fasting plasma glucose; PGI, pepsinogen I; PGII, pepsinogen II.

**Table 2:** Association Between MPO Tertiles and *H. pylori* Infection Risk or DPM Values.

	T2, (20.6, 31]	T1 <= 20.6		T3, >=31	
*H. pylori* infection		OR (95% CI)	*p* value	OR (95% CI)	*p* value
crude model	ref	1.15 (1.06, 1.25)	0.001	1.18 (1.08, 1.28)	<0.001
Model 1	ref	1.14 (1.05, 1.24)	0.002	1.19 (1.10, 1.30)	<0.0001
Model 2	ref	1.14 (1.05, 1.24)	0.002	1.19 (1.10, 1.30)	<0.0001
Model 3	ref	1.36 (1.24, 1.49)	<0.0001	1.12 (1.02, 1.22)	0.02
DPM		β (95% CI)	*p* value	β (95% CI)	*p* value
crude model	ref	22.45 (11.63, 33.27)	<0.0001	24.75 (13.91, 35.59)	<0.0001
Model 1	ref	22.87 (12.07, 33.67)	<0.0001	25.61 (14.78, 36.44)	<0.0001
Model 2	ref	22.41 (11.60, 33.22)	<0.0001	25.67 (14.84, 36.50)	<0.0001
Model 3	ref	37.1 (26.66, 47.53)	<0.0001	19.27 (8.81, 29.72)	<0.001

Model 1: adjust for Age, Sex; model 2: adjust for Age, Sex, BMI, Waist-to-hip ratio, Smoke and Drink; model 3: adjust for Age, Sex, BMI, Waist-to-hip ratio, Smoke, Drink, Diabetes, Hyperlipidemia, PGI/PGII, HDL, LDL, WBC, Platelet, Lymphocyte, Neutrophil and Monocyte.

**Table 3:** Threshold Effect Analysis of MPO on *H. pylori* Infection Risk.

	Crude Model	Model 1	Model 2	Model 3
	OR (95% CI) *p* Value	OR (95% CI) *p* Value	OR (95% CI) *p* Value	OR (95% CI) *p* Value
two-piecewise linear regression				
MPO < 24	0.983 (0.972, 0.994) 0.003	0.983 (0.972, 0.995) 0.004	0.983 (0.972, 0.995) 0.004	0.959 (0.947, 0.971) <0.0001
MPO ≥ 24	1.005 (1.003, 1.008) <0.0001	1.006 (1.003, 1.008) <0.0001	1.006 (1.003, 1.008) <0.0001	1.004 (1.002, 1.007) <0.001
*p* for Log-likelihood ratio	<0.0001	<0.0001	<0.0001	<0.0001

Model 1: adjust for Age, Sex; model 2: adjust for Age, Sex, BMI, Waist-to-hip ratio, Smoke and Drink; model 3: adjust for Age, Sex, BMI, Waist-to-hip ratio, Smoke, Drink, Diabetes, Hyperlipidemia, PGI/PGII, HDL, LDL, WBC, Platelet, Lymphocyte, Neutrophil and Monocyte.

**Table 4:** Threshold Effect Analysis of MPO on DPM Values.

	Crude Model	Model 1	Model 2	Model 3
	β (95% CI) *p* Value	β (95% CI) *p* Value	β (95% CI) *p* Value	β (95% CI) *p* Value
two-piecewise linear regression				
MPO < 24	−2.106 (−3.577, −0.635) 0.005	−2.12 (−3.586, −0.655) 0.005	−2.061 (−3.529, −0.593) 0.006	−4.164 (−5.586, −2.742) <0.0001
MPO ≥ 24	0.799 (0.494, 1.104) <0.0001	0.829 (0.524, 1.133) <0.0001	0.831 (0.526,1.135) <0.0001	0.658 (0.361, 0.956) <0.0001
*p* for Log-likelihood ratio	<0.0001	<0.0001	<0.0001	<0.0001

**Table 5:** Subgroup Analyses of the Association Between MPO Tertiles and *H. pylori* Infection Risk.

	T2, (20.6, 31]	T1, <=20.6	T3, >=31	*p* for Interaction
Age (years)				0.195
>=45	ref	1.197 (1.072, 1.336)	1.097 (0.976, 1.233)	
<45	ref	1.108 (0.967, 1.269)	1.237 (1.087, 1.407)	
Sex				0.694
Male	ref	1.197 (1.066, 1.346)	1.165 (1.033, 1.314)	
Female	ref	1.114 (0.983, 1.263)	1.151 (1.017, 1.303)	
BMI (kg/m^2^)				0.513
<24	ref	1.107 (0.987, 1.241)	1.135 (1.011, 1.273)	
[24, 28)	ref	1.257 (1.087, 1.454)	1.248 (1.076, 1.448)	
>=28	ref	1.115 (0.844, 1.474)	1.007 (0.764, 1.328)	
Smoke				0.223
Current	ref	1.091 (0.918, 1.296)	1.144 (0.959, 1.363)	
Never	ref	1.165 (1.052, 1.289)	1.130 (1.020, 1.251)	
Former	ref	1.547 (1.027, 2.346)	1.880 (1.228, 2.892)	
Drink				0.195
Current	ref	1.198 (1.057, 1.359)	1.146 (1.006, 1.306)	
Never	ref	1.117 (0.993, 1.256)	1.152 (1.026, 1.294)	
Former	ref	2.320 (0.762, 7.343)	4.074 (1.377, 13.022)	
Hypertension				0.056
No	ref	1.163 (1.065, 1.271)	1.185 (1.084, 1.296)	
Yes	ref	1.116 (0.806, 1.546)	0.839 (0.597, 1.177)	
Diabetes				0.375
No	ref	1.171 (1.074, 1.277)	1.163 (1.066, 1.269)	
Yes	ref	0.805 (0.471, 1.377)	0.998 (0.541, 1.838)	
Hyperlipidemia				0.36
No	ref	1.163 (1.067, 1.268)	1.166 (1.069, 1.272)	
Yes	ref	1.012 (0.502, 2.053)	0.592 (0.254, 1.327)	

Adjust for Age, Sex, BMI, Waist-to-hip ratio, Smoke, Drink, Diabetes, Hyperlipidemia, PGI/PGII, HDL, LDL, WBC, Platelet, Lymphocyte, Neutrophil and Monocyte except the stratification variable in each case.

**Table 6:** Subgroup Analyses of the Association Between MPO Tertiles and DPM Values.

	T2, (20.6, 31]	T1, <=20.6	T3, >=31	*p* for Interaction
Age (years)				0.182
>=45	ref	33.123 (18.761, 47.484)	24.826 (9.597, 40.055)	
<45	ref	11.202 (−5.376, 27.781)	20.864 (5.010, 36.719)	
Sex				0.678
Male	ref	20.562 (8.087, 33.036)	19.182 (6.337, 32.027)	
Female	ref	27.016 (8.711, 45.321)	26.613 (8.417, 44.808)	
BMI (kg/m^2^)				0.53
<24	ref	21.492 (6.172, 36.813)	24.471 (8.973, 39.970)	
[24, 28)	ref	29.142 (11.707, 46.576)	27.133 (9.311, 44.955)	
>=28	ref	16.546 (−12.135, 45.227)	−1.679 (−29.876, 26.517)	
Smoke				0.312
Current	ref	6.499 (−12.034, 25.033)	14.377 (−4.579, 33.333)	
Never	ref	27.181 (13.625, 40.738)	23.327 (9.686, 36.968)	
Former	ref	61.504 (19.426, 103.582)	59.337 (14.643, 104.031)	
Drink				0.456
Current	ref	17.424 (3.110, 31.739)	17.417 (2.557, 32.277)	
Never	ref	29.064 (13.027, 45.102)	26.298 (10.371, 42.226)	
Former	ref	18.875 (−71.539, 109.288)	99.605 (8.929, 190.281)	
Hypertension				0.252
No	ref	23.95 (12.667, 35.233)	24.594 (13.163, 36.026)	
Yes	ref	24.185 (−15.064, 63.433)	−4.765 (−44.505, 34.974)	
Diabetes				0.822
No	ref	24.392 (13.401, 35.382)	23.039 (11.939, 34.138)	
Yes	ref	14.365 (−54.467, 83.197)	14.451 (−65.562, 94.464)	
Hyperlipidemia				0.892
No	ref	24.23 (13.259, 35.200)	23.108 (12.013, 34.203)	
Yes	ref	15.5 (−54.624, 85.623)	−15.091 (−93.198, 63.017)	

## Data Availability

The health check-up data related to MPO used in our article are available from the corresponding author upon reasonable request.

## Supplementary Materials

The following supporting information can be downloaded at https://www.mdpi.com/article/10.3390/jcm14176019/s1, Figure S1: Differences in Key Characteristics Across MPO Tertiles; Table S1: Characteristics of the participants excluded from the analyses and those included in the final analyses; Table S2: Association Between MPO Tertiles and *H. pylori* Infection Risk or DPM Values in participants with normal inflammation status.
