# Supplementary material for: Nonlinear Relationship Between Myeloperoxidase Levels and Helicobacter pylori Infection Risk in Chinese Adults: A Population-Based Cross-Sectional Study
Source: J Clin Med. 2025 Aug 26;14(17):6019. doi: 10.3390/jcm14176019 (PMC12428885; doi:10.3390/jcm14176019)
Supplement: Supplementary file 1 [file jcm-14-06019-s001.zip › jcm-3752585-supplementary.pdf]

Nonlinear Relationship Between Myeloperoxidase Levels and *Helicobacter pylori* Infection Risk in Chinese Adults: A Population-Based Cross-Sectional Study

Junteng Zhou [1,†], Qihang Kong [2,†], Xiaojing Liu [2,3,*] and Yan Huang [1,4,5,6,*]

1 Health Management Center, General Practice Medical Center, West China Hospital, Sichuan University, Chengdu 610041, China; zhoujunteng@scu.edu.cn
2 Laboratory of Cardiovascular Diseases, Regenerative Medicine Research Center, West China Hospital, Sichuan University, Chengdu 610041, China; 2022324090006@stu.scu.edu.cn
3 Department of Cardiology, West China Hospital, Sichuan University, Chengdu 610041, China
4 State Key Laboratory of Respiratory Health and Multimorbidity, Chengdu 610041, China
5 Research Laboratory for Prediction and Evaluation of Chronic Diseases in the Elderly, National Clinical Research Center for Geriatric Diseases, Chengdu 610041, China
6 General Practice Research Institute, West China Hospital, Sichuan University, Chengdu 610041, China
* Correspondence: liuxq@scu.edu.cn (X.L.); yanhuang@wchscu.cn (Y.H.)
† These authors contributed equally to this work.

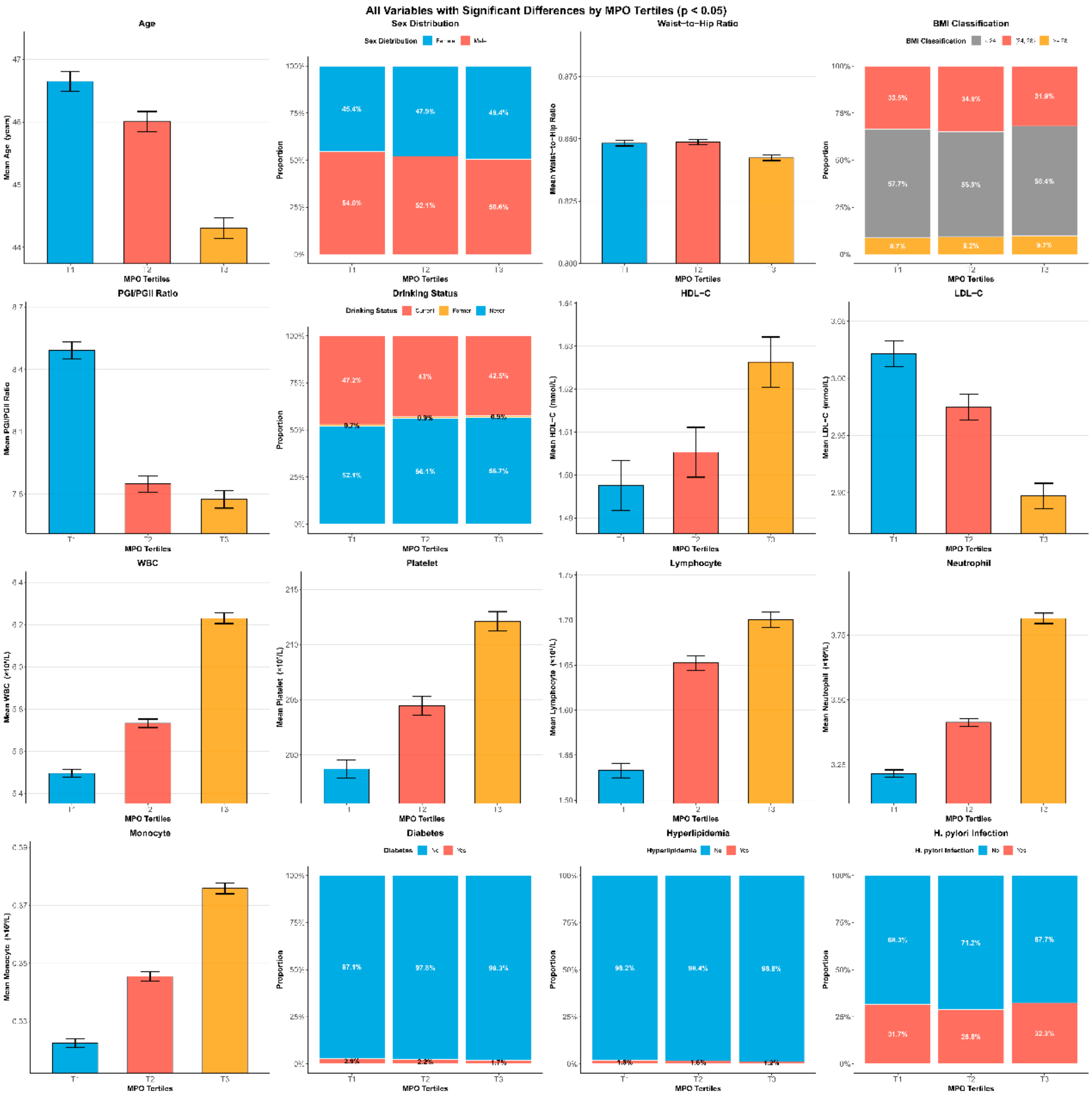

Figure S1 Differences in Key Characteristics Across MPO Tertiles

Table S1. Characteristics of the participants excluded from the analyses and those included in the final analyses

	Total (n=19920)	Excluded (n=4740)	Included (n=15180)
Age	45.65 ± 12.25	45.61 ± 14.37	45.66 ± 11.51
Sex			
Female	9501(47.70)	2278(48.06)	7223(47.58)
Male	10419(52.30)	2462(51.94)	7957(52.42)
BMI (kg/m2)			
< 24	6570(33.33)	1493(32.93)	5077(33.45)
[24, 28)	11352(57.58)	2643(58.29)	8709(57.37)
>= 28	1792(9.09)	398(8.78)	1394(9.18)

Waist-to-hip ratio	0.85 ± 0.08	0.84 ± 0.08	0.85 ± 0.08
Smoke			
Current	4464(22.41)	918(19.37)	3546(23.36)
Former	909(4.56)	242(5.11)	667(4.39)
Never	14547(73.03)	3580(75.53)	10967(72.25)
Drink			
Current	8485(42.60)	1772(37.38)	6713(44.22)
Former	170(0.85)	45(0.95)	125(0.82)
Never	11265(56.55)	2923(61.67)	8342(54.95)
Hypertension			
No	18336(92.05)	4132(87.17)	14204(93.57)
Yes	1584(7.95)	608(12.83)	976(6.43)
Diabetes			
No	19238(96.58)	4405(92.93)	14833(97.71)
Yes	682(3.42)	335(7.07)	347(2.29)
Hyperlipidemia			
No	19592(98.35)	4648(98.06)	14944(98.45)
Yes	328(1.65)	92(1.94)	236(1.55)
AST(U/L)	21.00(17.00,25.00)	21.00(17.00,25.00)	21.00(17.00,26.00)
ALT (U/L)	20.00(14.00,29.00)	19.00(13.00,28.00)	20.00(14.00,30.00)
GGT (U/L)	22.00(14.00,37.00)	21.00(14.00,35.00)	22.00(14.00,38.00)
PGI/PGII	8.06 ± 2.92	8.18 ± 3.06	8.04 ± 2.90
HDL-C (mg/dL)	1.51 ± 0.42	1.50 ± 0.42	1.51 ± 0.42
LDL-C (mg/dL)	2.95 ± 0.81	2.92 ± 0.82	2.96 ± 0.80
WBC ($\times 10^9$/L)	5.86 ± 1.58	6.00 ± 1.61	5.82 ± 1.56
Platelet ($\times 10^9$/L)	207.17 ± 60.43	213.94 ± 61.12	205.09 ± 60.06
Lymphocyte ($\times 10^9$/L)	1.66 ± 0.62	1.77 ± 0.72	1.63 ± 0.59
Neutrophil ($\times 10^9$/L)	3.50 ± 1.23	3.58 ± 1.24	3.48 ± 1.23
Monocyte ($\times 10^9$/L)	0.35 ± 0.12	0.36 ± 0.12	0.35 ± 0.12
MPO (ng/ml)	31.68 ± 26.96	32.87 ± 26.86	31.31 ± 26.98
DPM	35.00(0.00,150.00)	35.00(0.00,130.00)	35.00(0.00,150.00)
H. pylori infection			
No	11994(69.21)	1513(70.37)	10481(69.04)
Yes	5336(30.79)	637(29.63)	4699(30.96)

Table S2. Association Between MPO Tertiles and *H. pylori* Infection Risk or DPM Values in participants with normal inflammation status

	**T2, (20.6,31]**	**T1 <=20.6**		**T3, >=31**	
H. pylori infection		OR (95%CI)	p value	OR (95%CI)	p value
crude model	ref	1.19(1.09,1.29)	<0.001	1.18(1.08,1.29)	<0.001

Model 1	ref	1.18(1.08,1.29)	<0.001	1.19(1.09,1.30)	<0.0001
Model 2	ref	1.18(1.08,1.29)	<0.001	1.19(1.09,1.30)	<0.0001
Model 3	ref	1.4(1.27,1.54)	<0.0001	1.12(1.02,1.23)	0.02
DPM		β (95%CI)	p value	β (95%CI)	p value
crude model	ref	24.89(13.67,36.10)	<0.0001	25.07(13.85,36.29)	<0.0001
Model 1	ref	25.35(14.16, 36.54)	<0.0001	25.37(14.15, 36.58)	<0.0001
Model 2	ref	24.88(13.68, 36.09)	<0.0001	25.41(14.19, 36.62)	<0.0001
Model 3	ref	39.32(28.53, 50.12)	<0.0001	18.98(8.22, 29.75)	<0.001

Model 1: adjust for Age, Sex, model 2: adjust for Age, Sex, BMI, Waist-to-hip ratio, Smoke and Drink, model 3: adjust for Age, Sex, BMI, Waist-to-hip ratio, Smoke, Drink, Diabetes, Hyperlipidemia, PGI/PGII, HDL, LDL, WBC, Platelet, Lymphocyte, Neutrophil and Monocyte
